# Geographical variations in bacterial communities associated with soft coral *Scleronephthya gracillimum*

**DOI:** 10.1371/journal.pone.0183663

**Published:** 2017-08-31

**Authors:** Seonock Woo, Shan-Hua Yang, Hsing-Ju Chen, Yu-Fang Tseng, Sung-Jin Hwang, Stephane De Palmas, Vianney Denis, Yukimitsu Imahara, Fumihito Iwase, Seungshic Yum, Sen-Lin Tang

**Affiliations:** 1 Korea Institute of Ocean Science & Technology, Geoje, Republic of Korea; 2 Faculty of Marine Environmental Science, University of Science and Technology (UST), Geoje, Republic of Korea; 3 Biodiversity Research Center, Academia Sinica, Taipei, Taiwan; 4 Department of Eco-Biological Science, Woosuk University, Jincheon, Republic of Korea; 5 Biodiversity Program, Taiwan International Graduate Program, Academia Sinica and National Taiwan Normal University, Taipei, Taiwan; 6 Department of Life Science, National Taiwan Normal University, Taipei, Taiwan; 7 Institute of Oceanography, National Taiwan University, Taipei, Taiwan; 8 Wakayama Laboratory, Biological Institute on Kuroshio, Wakayama City, Wakayama, Japan; 9 Shikoku Marine Life Laboratory, Otsuki-Town, Kochi, Japan; National Cheng Kung University, TAIWAN

## Abstract

Environmental impacts can alter relationships between a coral and its symbiotic microbial community. Furthermore, changes in the microbial community associated with increased seawater temperatures can cause opportunistic infections, coral disease and death. Interactions between soft corals and their associated microbes are not well understood. The species *Scleronephthya gracillimum* is distributed in tropical to temperate zones in coral assemblages along the Kuroshio Current region. In this study we collected *S*. *gracillimum* from various sites at different latitudes, and compared composition of their bacterial communities using Next Generation Sequencing. Coral samples from six geographically distinct areas (two sites each in Taiwan, Japan, and Korea) had considerable variation in their associated bacterial communities and diversity. Endozoicimonaceae was the dominant group in corals from Korea and Japan, whereas *Mycoplasma* was dominant in corals from Taiwan corals. Interestingly, the latter corals had lower relative abundance of Endozoicimonaceae, but greater diversity. These biogeographic differences in bacterial composition may have been due to varying environmental conditions among study locations, or because of host responses to prevailing environmental conditions. This study provided a baseline for future studies of soft coral microbiomes, and assessment of functions of host metabolites and soft coral holobionts.

## Introduction

Coral ecosystems are among the most biologically diverse on Earth, with large reef-building corals in tropical and subtropical waters providing important habitats for numerous marine organisms [1–2]. Coral species are well adapted to short-term natural events, but long-term stresses (e.g. climate change and human-induced stresses) can cause coral bleaching and death [3]. In addition, it is thought that coral-associated microorganisms are important to coral nutrition, fitness and survival [4]. These symbiotic microbes, which include dinoflagellates, bacteria, viruses and archaea, are also sensitive to environmental perturbations and the physiological status of their coral hosts [5–6]. Consequently, characterizing relationships among environmental conditions, patterns of microbial communities and occurrence of coral disease is of fundamental importance for reef conservation.

Most studies of coral-associated bacteria have focused on reef building corals (Scleractinia), with relatively few studies of the microbiomes of octocorals, and those focussed on members of the Alcyonacea [7–12]. Among studies of Alcyonacea-associated microbiomes, the bacterial genus *Endozoicomonas* (Gammaproteobacteria) has been reported to be the dominant bacterial group in various host species [7–12]. It is noteworthy there have been reports of other bacteria being present in these microbiomes, including members of the genera Vibrionaceae, Tenericute, and Alphaproteobacteria [13–14]. Various environmental factors that differ among locations and seasons, including temperature and nutrients, influenced the microbial composition of stony corals [15–18]. In previous studies of microbiomes in Alcyonacea, samples were usually collected from single or nearby sites (usually within 2–3° latitude), making it difficult to determine whether differences in microbial composition of soft coral tissues were caused by environmental influences resulting from latitudinal differences.

The Kuroshio Current (KC) is a warm current that is subject to rapid increases in temperature due to global climate change [19]. In this regard, the current warmed most rapidly in the period 1981–1998, when surface temperatures rose by 1.5°C (0.9°C/decade, almost 7 times the global rate) [20]. As the KC runs from the tropical Philippines past subtropical Taiwan to the temperate region of Japan, it transfers heat from lower to higher latitudes [21–24]. Because the KC is warming rapidly, some reef-building corals [25], numerous fish species [26] and other animals [27] in the region may expand their ranges northward in response to increasing temperatures [25]. Furthermore, coral reefs of Taiwan and Japan were closely linked by the KC [28], including some soft corals (e.g. Alcyoniidae, Nephtheidae and Xeniidae).

Most corals in Korea are soft corals and gorgonians in the order Alcyonacea, subclass Octocorallia. Among the 156 coral species reported from Korea, 89 were classified as Octocorallia and 67 as Hexacorallia [29–30]. The number of octocoral species reported from Japan is 643 [31], with 69 species reported from southern Taiwan [32]. Among the soft corals, *Scleronephthya gracillimum* is common in Korea (Jeju Island), Japan and Taiwan. At Jeju Island in Korea, *S*. *gracillimum* is a dominant species among benthic assemblages [33].

*S*. *gracillimum* is an azooxanthellate coral typically present at depths of 15–40 m around Japan, Korea and Taiwan. This soft coral species is of biochemical and pharmacological importance, as it contains bioactive steroids having cytotoxic and antiviral activities [34]. Therefore, characterizing bacterial composition of *S*. *gracillimum* is essential for understanding the microbiome of the coral, and for assessing functions of host metabolites and the soft coral holobiont. Thus, this study focused on characterization of the bacterial communities among specimens of *S*. *gracillimum* collected at various latitudes along the path of the KC.

## Materials and methods

### Sample sites and collections

**Fig 1:**
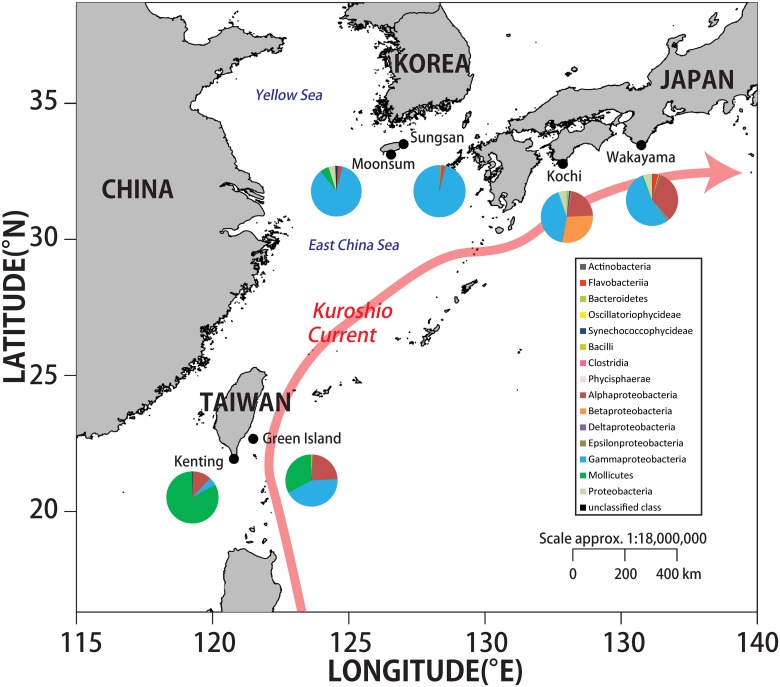
Relative abundance of major bacterial taxa in the soft coral *Scleronephthya gracillimum* from various locations. Each pie chart indicates bacterial composition in *S*. *gracillimum* from a specific location. The map was created in R 3.2.3 (R Core Team 2015; https://www.R-project.org) using the maptools package and spatial data freely available on the DIVA-GIS website (http://www.diva-gis.org/Data).

Two sites were selected in each country (Japan, Taiwan and Korea; Fig 1 and Table 1). Ten or 12 colonies of healthy *S*. *gracillimum* were collected from each site, following identification in situ. The sample colony size ranged from 12–15 cm including the extended polys. Each specimen was collected in a zipper bag, and processed on the boat immediately after collection. Each sample was individually wrapped in foil, and samples were preserved at –80°C for transport in a Taylor-Wharton’s CX100 Dry Shipper (Taylor-Wharton; AL, USA). Sampling was performed in May and June 2012, except for the KRM site in Korea. The target species *S*. *gracillimum* in this study is not listed as endangered species. Samples were not collected from national parks or natural reserves in Japan, thus no specific permission was required for sampling this species in Japan and the sampling in Korea was under the permission of Korean Coral Resources Bank (KCRB) by the Ministry of Land, Transport and Maritime Affairs, Korea. The sampling in Taiwan was under the permission number 1010146189 issued by the Taitung County Government and permission number 1010001032 issued by the Kenting National Park. A map was created in R 3.2.3 (R Core Team 2015; https://www.R-project.org) [35] using the maptools package [36] and spatial data freely available on the DIVA-GIS website (http://www.diva-gis.org/Data).

**Table 1:** *Scleronephthya gracillimum* sampling information and *S*. *gracillimum*- associated microbial diversity indices.

	JPK	JPW	KRM	KRS	TWG	TWK
Location (GPS)	Kochi, Japan; 32.78°N, 132.86°E	Wakayama, Japan; 33.48°N, 135.73°E	Moonsum, Korea; 33.23°N, 126.57°E	Sungsan, Korea; 33.46°N, 126.95°E	Green Island, Taiwan; 22.67°N, 121.50°E	Kenting, Taiwan; 21.56°N, 120.45°E
Collecting date	May 7, 2012	May 13, 2012	Jun 21, 2012	Feb 10, 2012	Jun 06, 2012	Jun 03, 2012
Depth (m)	13	22	14	22	15	17
No. of samples	12	12	10	10	12	12
Temperature range (°C)	16.4–27.8		15.7–26.1		24.9–28.2	
Total sequence	3562	3912	740	5532	10299	1400
OTUs^a^	60	55	19	22	43	26
Richness	7.21	6.53	2.72	2.44	4.55	3.45
Evenness	0.64	0.67	0.64	0.39	0.62	0.28
Shannon	2.62	2.67	1.89	1.19	2.33	0.92
Simpson^b^	0.87	0.85	0.78	0.58	0.85	0.31

^a^Operational taxonomic units

^b^Simpson's Index of diversity

### DNA extraction

Soft coral polyp tissues were mortar-pulverized in liquid nitrogen and the powder homogenized in lysis solution [60 mM Tris (pH 8.0), 10 mM EDTA, 0.5% SDS], incubated at 37°C for 1 h after RNase A, proteinase K (500 ug/ml) was added, and incubated at 50°C for 1 h. The mixture was centrifuged at 10,000 rpm for 30 min at 20°C, and the aqueous phase moved to a new tube. The DNA was extracted (three times with a phenol:chloroform mixture, followed by chloroform per se, with separating phases centrifuged at 10,000 rpm for 15 min at 20°C). The 1/10 volume of 3 M sodium acetate (pH 5.2) and the same volume of 95% ethanol were added into the retained aqueous phase. Precipitated DNA was washed using 70% ethanol and re-suspended in Tris-EDTA buffer (pH 7.4). For library construction, DNA were extracted from individual colonies in each site then pooled (with equal amounts of DNA from each site).

### Library construction and Next Generation Sequencing

A library was prepared using PCR products, according to the GS FLX titanium library prep guide. Libraries were quantified using Picogreen assay (Promega, WI, USA). The emPCR, corresponding to clonal amplification of the purified library, was done using the GS-FLX titanium emPCR Kit (454 Life Sciences, CT, USA). Emulsion was dispensed into a 96-well plate and the PCR amplification program was run according to the manufacturer's recommendations. A 20 ng aliquot of each sample DNA was used for a 50 ul PCR reaction. The 16S universal primers 27F (5’ GAGTTTGATCMTGGCTCAG 3’) and 800R (5’ TACCAGGGTATCTAATCC 3’) [37] were used to amplify 16S rRNA genes. FastStart High Fidelity PCR System (Roche, Basel, Switzerland) was used for PCR under the following conditions: 94°C for 3 min followed by 35 cycles of 94°C for 15 s; 55°C for 45 s and 72°C for 1 min; and a final elongation step at 72°C for 8 min. After the PCR reaction, products were purified using AMPure beads (Beckman Coulter, CA, USA).

Sequencing was done by Macrogen Ltd. (Seoul, Korea) using Genome Sequencer FLX titanium (454 Life Sciences, CT, USA) following PCR amplification. Each sample was loaded in 1 region of a 70–75 mm PicoTiter plate (454 Life Sciences, CT, USA) fitted with an 8-lane gasket. Pyrosequencing data were deposited in the National Center for Biotechnology Information Sequence Read Archive (accession number SRP083934).

### Data analyses

To survey bacterial communities in *S*. *gracillimum* from various locations, V1-V4 sequence reads were sorted and trimmed (qscore = 27, maxhomop = 8, maxambig = 0) by MOTHUR software [38]. Furthermore, filtered sequences were subjected to chimera removal by USEARCH (using Ribosomal database project (RDP) gold database). The operational taxonomic units OTUs were identified from remaining sequences using USEARCH (UPARSE) [39] at 3% divergence value and were classified by a greengenes database. Rarefied diversity measures were estimated using MOTHUR software with 1000 iterations. Sample size (sequence numbers) varied from 740 to 10299; thus, 740 was chosen for rarefaction analysis. Following data normalization, Bray-Curtis similarities were calculated by Primer 6 [40] based on relative abundances of each bacterial class for each location. Farthest neighbor clustering (complete linkage) was used to visualize relationships of microbial communities among various locations. Venn diagrams were presented by Venny 2.0 (http://bioinfogp.cnb.csic.es/tools/venny/index.html). Evolutionary analyses were conducted in MEGA7 [41].

## Results

High-throughput 454 pyrosequencing of the hypervariable V1 to V4 regions of bacterial 16S ribosomal RNA was done to investigate bacterial composition in the tissues of *S*. *gracillimum* specimens collected from Japan, Korea and Taiwan. In total, 25445 sequences were obtained, with the number of sequences for each sample ranging from 740 (KRM) to 10299 (TWG), and the number of operational taxonomic units (OTUs) ranging from 19 (KRM) to 60 (JPK) (Table 1). The Shannon’s index for the *S*. *gracillimum* samples ranged from 0.92 (TWK) to 2.67 (JPW), and the Simpson's Index of diversity ranged from 0.31 (TWK) to 0.87 (JPK). The Shannon’s index values for samples from the two locations in Japan were higher than those for the samples from Korea and Taiwan (Table 1).

A total of 102 bacterial OTUs were obtained from *S*. *gracillimum*. Among these, 94 OTUs were classified to the 13 classes and eight OTUs were unclassified classes (Fig 1). In the classes, Gammaproteobacteria (41 OTUs) and Alphaproteobacteria (33 OTUs) had most OTUs, whereas each of other classes had no more than five OTUs. With respect to relative abundance, Gammaproteobacteria was the dominant group in samples from each of the two sites in Korea (Moonsum: 82.27%; Sungsan: 96.17%) and Japan (Kochi: 41.21%; Wakayama: 53.86%), and also at Green Island in Taiwan (43.10%). Among the Gammaproteobacteria, the relative abundance of the family Endozoicimonaceae was high at all locations (S1 Fig). Alphaproteobacteria was also common in the Japanese and Taiwanese samples. However, the Alphaproteobacteria orders differed between the Japanese and Taiwanese samples: Rhodobacterales dominated in the former, whereas Richettsiales dominated in the latter (S1 Fig). In addition, unlike the samples from Japan and Korea, *Mycoplasma* (Mollicutes) dominated in the Taiwanese samples (Fig 1). In addition, an OTU that belonged to the genus *Ralstonia* was only present in the Kochi samples (Japan) (Fig 1), with a relative abundance up to 28.5%.

**Fig 2:**
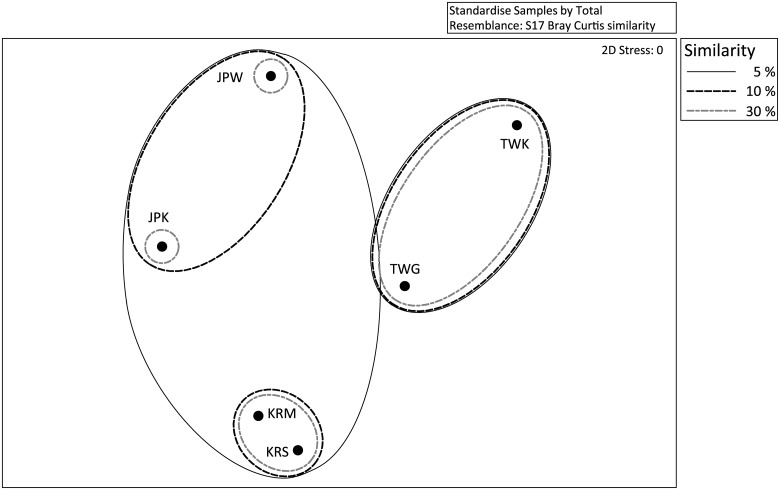
Non-metric multidimensional scaling (nMDS) plot analysis for *S*. *gracillimum*-associated samples from various locations. The Bray—Curtis similarity index was calculated using the relative percentage of each class in each sample, and nMDS ordination was performed in PRIMER 6. The *S*. *gracillimum*-associated bacterial composition is shown by site: TWG (Green Island, Taiwan), TWK (Kenting, Taiwan), KRM (Moonsum, Korea), KRS (Sungsan, Korea), JPK (Kochi, Japan) and JPW (Wakayama, Japan).

Based on the nMDS result (Fig 2 and S2 Fig), *S*. *gracillimum*-associated bacterial communities differed geographically. However, the communities in samples from locations where the latitude was < 1° clustered together, namely the two samples from Korea were most similar to each other (similarity: 70.8%); the two samples from Taiwan clustered together (similarity: 33.3%); and the two samples from Japan had 13.2% similarity. Bacterial communities tended to be grouped by site (Korea, Japan and Taiwan), but communities in Korean samples grouped more closely to samples from Japan than to those from Taiwan. However, based on square root and fourth root data transformations, Taiwan samples grouped more closely to samples from Japan than to those from Korea (Fig 2 and S2 Fig). Therefore, there were geographical differences in *S*. *gracillimum*-associated bacterial communities.

**Fig 3:**
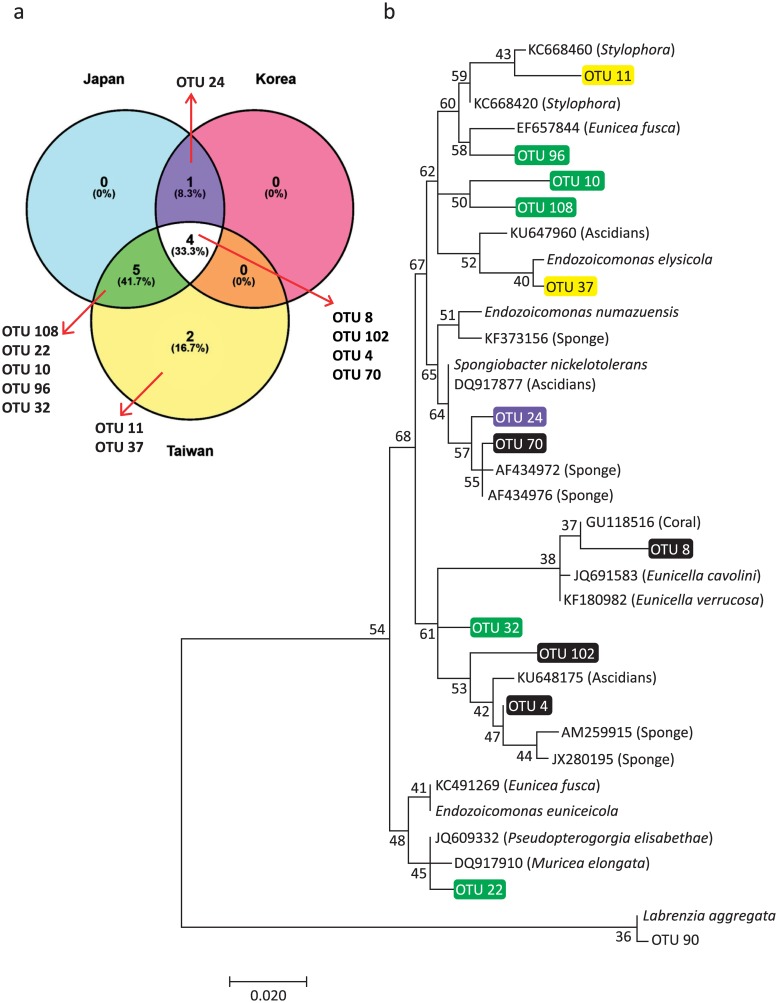
Venn diagrams and maximum likelihood tree for OTUs of Endozoicimonaceae associated with *S*. *gracillimum* from Korea, Japan and Taiwan. (a) Venn diagrams of Endozoicimonaceae in *S*. *gracillimum* from Korea, Japan and Taiwan. (b) The tree for the heuristic search was obtained by applying neighbor-join and BioNJ algorithms to a matrix of pairwise distances estimated using the Maximum Composite Likelihood (MCL) approach. The analysis involved 35 sequences, and included the outgroup *Labrenzia aggregate* and OTU90. Evolutionary analyses were conducted in MEGA7. The colours and associations of OTUs with countries are as follows: green for Taiwan and Japan; purple for Japan and Korea; and black for Korea, Japan and Taiwan. The origins of the sequences are shown in parentheses.

A total of 14 OTUs were present in samples from all three locations (core members). Relative abundance of core members was highest in Korean samples (> 80%) and lowest in the Taiwanese samples (< 20%; S3 Fig). In addition, four OTUs (OTU4, OTU8, OTU70 and OTU102) belonging to Endozoicimonanceae were dominant core members in Korean and Japanese samples (S3 Fig). Although Endozoicimonaceae were not dominant in Taiwanese samples, the number of Endozoicimonaceae OTUs was highest in these samples (Fig 3a), comprising 11 of the total 12 Endozoicimonaceae OTUs. Among these, two Endozoicimonaceae OTUs were present only in the Taiwanese samples, indicating geographical specificity (Fig 3a). Based on phylogenetic analysis, distribution of Endozoicimonaceae OTUs were associated with diverse bacteria species derived from different hosts, with no apparent phylogenetic relationship among OTUs (Fig 3b).

## Discussion

Microbial composition of soft corals has been considered stable, with *Endozoicomonas* being the dominant genus of bacteria in most healthy soft Octocorallia corals, e.g. gorgonians [7–10, 12] and *Sarcophyton* [42]. However, in the present study, bacterial composition of *S*. *gracillimum* varied among locations, with substantial differences occurring as a function of latitude, and with *Endozoicomonas* not consistently dominating the community. In contrast, *Mycoplasma* (class Mollicutes) and *Ehrlichia* (class Alphaproteobacteria) were relatively highly abundant in samples from Taiwan and Japan, respectively. Although Endozoicimonaceae was a core dominant member in Taiwanese and Japanese samples, geographical differences also occurred among core members, including differences in core Endozoicimonaceae members. In previous studies, distances among sampling sites did not exceed 10° latitude [7–14], which may not have been a sufficiently large separation to detect geographical differences in microbial composition. However, in the present study, latitudinal separation of sampling sites exceeded 10° (Taiwan to Japan: 11.92°; Taiwan to Korea: 11.90°). We inferred that large latitudinal differences or differences between oceans should be considered when investigating geographical aspects of microbial composition of soft corals.

Composition of coral-associated bacteria is known to be influenced by temperature and season [17]. Furthermore, seasonal changes in seawater temperature at Jeju Island and Japan (> 10°C) are larger than those for Taiwan (Table 1). Among the six sampling occasions, five occurred in the May–June period, whereas the KRS site was sampled in February. Although the KRM and KRS sites were sampled in very different seasons (June and February, respectively), based on clustering, there was a high degree of similarity between microbial communities from KRM and KRS samples. Thus, seasonal differences in Korea may be smaller than geographical differences. Consequently, temperature variations at different locations may affect the dominant bacteria in the soft coral *S*. *gracillimum*.

In addition to latitude, properties of currents could also have affected diversity [26]. In the present study, six sampling sites are affected and connected by the KC. However, different sites have different influences by seasonal variations of the KC and coastal currents. For example, Green Island is affected by the KC constantly, but Kenting area is affected by both the KC and the South China Sea [43], whereas in Jeju Island, Tsushima Current (a branch of the Kuroshio Current) is affected by the estuary of Hunaghe [44]. Thus, although warm temperature of the KC might lead to increased biodiversity [26], which may explain the result of higher bacterial diversity in TWG, JPK and JPW, various properties of the currents could also have contributed to locational differences in soft-coral-associated microbial composition; this possibility should be confirmed with additional and ideally long-term monitoring.

It has been reported that human activities, such as tourism, cause changes in composition of reef microbe communities [45], and bacterial diversity associated with octocorals was sensitive to anthropogenic disturbances [10]. In this study, our sampling sites are tourist destinations and have other anthropogenic disturbances including agriculture, land use, overfishing. However, we did not establish any relationship between bacterial diversity in *S*. *gracillimum* and human activities; therefore, further studies will be needed to investigate this phenomenon.

Although seawater sampling was not undertaken at the six sampling sites in this study, there have been several reports of the bacterial composition of seawater from some of these sites, or nearby. Bacterial compositions of seawater reported previously were different from that in *S*. *gracillimum*: in seawater of Korea, the dominant bacterial groups were Psedoalteromonadaceae, Vibrionaceae and SAR11-1 [46]; in Green Island and Kenting, *Vibrio*, *Cyanobacteria*, and Marin group A were majority [47–48]. Although bacterial composition of seawater is different from that of *S*. *gracillimum*, it was unclear if minor bacterial groups in seawater become soft coral-associated bacteria, influencing the bacterial composition of *S*. *gracillimum*. Hence, further studies comparing microbial composition in relevant seawater and *S*. *gracillimum* would be useful to understand formation of the soft coral holobiont.

Variations in reproductive strategy among coral hosts may also affect bacterial composition in hexacorals. For example, microbial composition in *Stylophora pistillata* (a brooder coral) were strongly clustered on the basis of geographical location, which had been suggested that *S*. *pistillata* may vertically transfer microbes from parents to offspring [49], controlling the microbial structure and development, and this may result in geographic grouping [50]. However, grouping was weaker in the broadcast spawner *Pocillopora verrucosa* [50], as *P*. *verrucosa* larvae obtain a mixture of microbes associated with seawater passing over distinct reefs [51]. Unlike the two hexacorals, in this study, *S*. *gracillimum* is a broadcast species [52], but its bacterial composition had geographical differences. If the reproductive strategy of a coral host affects its microbial composition, gametes of *S*. *gracillimum* in different locations might acquire distinct microorganisms from those locations, and so develop communities shaped by local environmental conditions. Therefore, additional factors may influence bacterial communities in soft corals. Future studies of soft coral-associated microbes should consider host reproductive strategies, host metabolites and environmental factors.

Alphaproteobacteria dominate in invertebrates lacking photosynthetic symbionts, whereas Gammaproteobacteria dominate in those hosting photosynthetic symbionts [53]. Furthermore, the presence of *Symbiodinium* can affect the abundance of Endozoicimonaceae (class Gammaproteobacteria) in stony corals [18] and gorgonians [11]. Relative abundance of Endozoicimonaceae is higher in hosts harbouring zooxanthellae [11]. However, in the present study, Endozoicimonaceae dominated in the azooxanthellate coral *S*. *gracillimum*, and relative abundance of its members varied among locations. Hence, we inferred that locational factors might also affect the abundance of *Endozoicomonas* and other Gammaproteobacteria in Cnidaria, in addition to photosynthetic symbionts.

*Endozoicomonas* genotypes can have diverse roles in different hosts, from symbiotic [9] to parasitic [54]. Endozoicimonaceae belong to the order Oceanospirillales, an aerobic heterotrophic group having the ability to degrade complex organic compounds produced by the host or symbiotic algae [55]. In addition, some species of *Endozoicomonas* are able to completely degrade testosterone from corals for recognizing their host [56]. *Endozoicomonas* is frequently associated with soft corals, and many soft corals, including *S*. *gracillimum*, are rich sources of steroids [34]. Our finding that *Endozoicomonas* was a dominant bacterial group in *S*. *gracillimum* suggests that the metabolism of soft coral steroids by *Endozoicomonas* may be worthy of further investigation.

Members of the Betaproteobacteria were generally uncommon in samples from the study sites, although an OTU that belonged to *Ralstonia* was dominant at the Kochi site in Japan. *Ralstonia* phylotypes have been routinely reported to have a relative abundance of < 2–5% in stony corals and gorgonians [57–58], and have been regarded as relatively rare members [59]. Because of their low abundance in corals, the function of *Ralstonia* has not been considered until recently. *Ralstonia* may have a function related to nitrogen fixation, through its symbiotic relationship with plants [59]. Although most studies have focused on scleractinian corals, nitrogen cycling (including nitrogen fixation) has recently been reported in soft corals, and is now considered essential in coral holobionts [11, 60].

*Mycoplasma* (phylum Tenericute) has been detected in stony corals and gorgonians from both deep sea and shallow water sites [14, 61–62]. Therefore, although *Mycoplasma* has not been regarded as a core component of scleractinian corals, it is regarded as a potential key albeit understudied component of the microbiome in gorgonians [14]. *Mycoplasma* is distinct from other bacteria because it is smaller and cells lack a cell wall [63]. Many mycoplasmas are pathogens that rely on their host to supply nutrients that they lack the capacity to biosynthesize [64–65]. However, the mycoplasmas in corals may be commensal rather than parasitic. *Mycoplasma* cells occur in the cnidocysts of corals, and it has been suggested that they assimilate amino acids and fatty acids leaking from prey during coral host feeding; furthermore, it has been suggested that these bacteria may be harmless to their hosts [62]. In this study, *Mycoplasma* only dominated in the Taiwan samples, and the *Mycoplasma* phylotypes differed among the three study locations. The two sampling sites in Taiwan represented highly biodiverse marine ecosystems [66], and have recently been subject to the impacts of expanded tourism [67]. As hundreds of *Mycoplasma* species can infect animals, including humans [68], human activities at the two sites may have resulted in the relatively high abundance of *Mycoplasma* in *S*. *gracillimum* at these sites.

The Alphaproteobacteria in the samples from Japan and Taiwan differed between these study sites. Although Rhodobacterales was a core member at the three locations, its relative abundance was less than that of Richettsiales in Taiwan samples. Rhodobacterales are ubiquitous in coral reef systems, including surface waters, reef invertebrates, and stony and soft corals [9, 45, 49]. Rickettsiales are common in tropical corals, and are well known pathogens of marine invertebrates [69]. Because their functional characteristics are generally influenced by temperature and light intensity, Lawler et al. [11] suggested that Rickettsiales in gorgonians have different expressions of opportunistic inclination in tropical versus cold-water environments. It may be that the high abundance of Rickettsiales in our study was a consequence of higher seawater temperatures in Taiwan.

## Conclusion

In this study, bacterial communities associated with the soft coral *S*. *gracillimum* were differed among study sites in Korea, Japan and Taiwan. These study sites were all affected by the Kuroshio Current, which is one of the world’s fastest currents and transfers heat from equatorial regions to higher latitudes. Large distances between sites probably resulted in differing seawater properties, and may have affected bacterial community composition. There were geographical differences in dominant bacteria among sites, particularly occurrence of Oceanospirillales (Endozoicimonaceae), Rickettsiales, Rhodobacterales, and the genera *Ralstonia* and *Mycoplasma*. This was apparently the first report indicating geographical differences in the bacterial communities in *S*. *gracillimum*. Futhermore, we also detected occurrence of core bacterial members in this soft coral, including Endozoicimonaceae and Rhodobacteraceae, which indicates that these bacteria have an intimate relationship with their host. Further studies will be necessary to clarify the relationships among *S*. *gracillimum*, host metabolites, and the symbiotic microbial communities and their function.

## Supporting information

S1 Fig Relative abundance of sub-taxa in *Gammaproteobacteria* and *Alphaproteobacteria* associated with *S*. *gracillimum* in each location. Colors indicate sub-taxa. The *S*. *gracillimum* associated bacterial composition were denoted as TWG (Green Island, Taiwan), TWK (Kenting, Taiwan), KRM (Moonsum, Korea), KRS (Sungsan, Korea), JPK (Kochi, Japan) and JPW (Wakayama, Japan).
(DOCX)

S2 Fig The nMDS and clustering result of bacterial composition in *S*. *gracillimum* from six sampling sites. (a) and (b) are nMDS and cluster analysis of data with square root transformation. (c) and (d) are nMDS and cluster analysis of data with fourth root transformation.
(DOCX)

S3 Fig The relative abundance of 14 core members in each location. Colors indicate OTUs. The *S*. *gracillimum* associated bacterial composition were denoted as TWG (Green Island, Taiwan), TWK (Kenting, Taiwan), KRM (Moonsum, Korea), KRS (Sungsan, Korea), JPK (Kochi, Japan) and JPW (Wakayama, Japan).
(DOCX)
