# Supplementary material for: Geographical variations in bacterial communities associated with soft coral Scleronephthya gracillimum
Source: PLoS One. 2017 Aug 31;12(8):e0183663. doi: 10.1371/journal.pone.0183663 (PMC5578639; doi:10.1371/journal.pone.0183663)
Supplement: S1 Fig — Colors indicate sub-taxa. The S. gracillimum associated bacterial composition were denoted as TWG (Green Island, Taiwan), TWK (Kenting, Taiwan), KRM (Moonsum, Korea), KRS (Sungsan, Korea), JPK (Kochi, Japan) and JPW (Wakayama, Japan). (DOCX) [file pone.0183663.s001.docx]

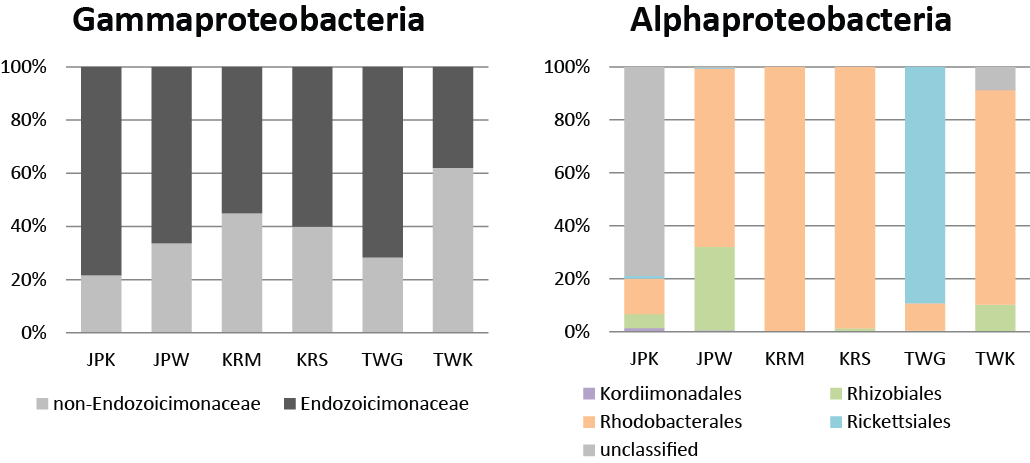


**Figure S1. Relative abundance of sub-taxa in *Gammaproteobacteria* and *Alphaproteobacteria* associated with *S. gracillimum* in each location.**
