# Supplementary material for: Geographical variations in bacterial communities associated with soft coral Scleronephthya gracillimum
Source: PLoS One. 2017 Aug 31;12(8):e0183663. doi: 10.1371/journal.pone.0183663 (PMC5578639; doi:10.1371/journal.pone.0183663)
Supplement: S2 Fig — (a) and (b) are nMDS and cluster analysis of data with square root transformation. (c) and (d) are nMDS and cluster analysis of data with fourth root transformation. (DOCX) [file pone.0183663.s002.docx]

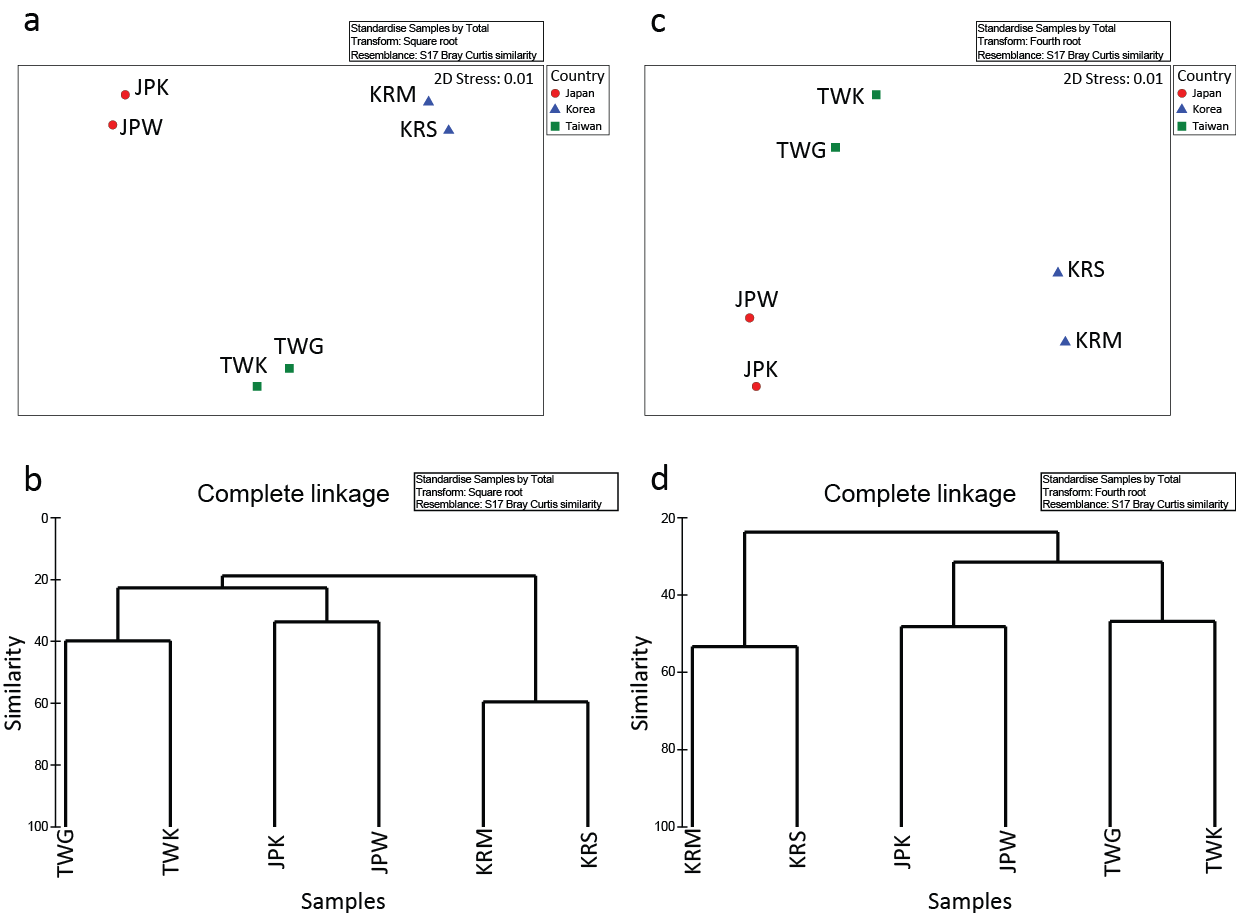


**Figure S2. The nMDS and clustering result of bacterial composition in *S. gracillimum* from six sampling sites.**
