# Supplementary figures and images for: Geographical variations in bacterial communities associated with soft coral Scleronephthya gracillimum
Source: PLoS One. 2017 Aug 31;12(8):e0183663. doi: 10.1371/journal.pone.0183663 (PMC5578639; doi:10.1371/journal.pone.0183663)

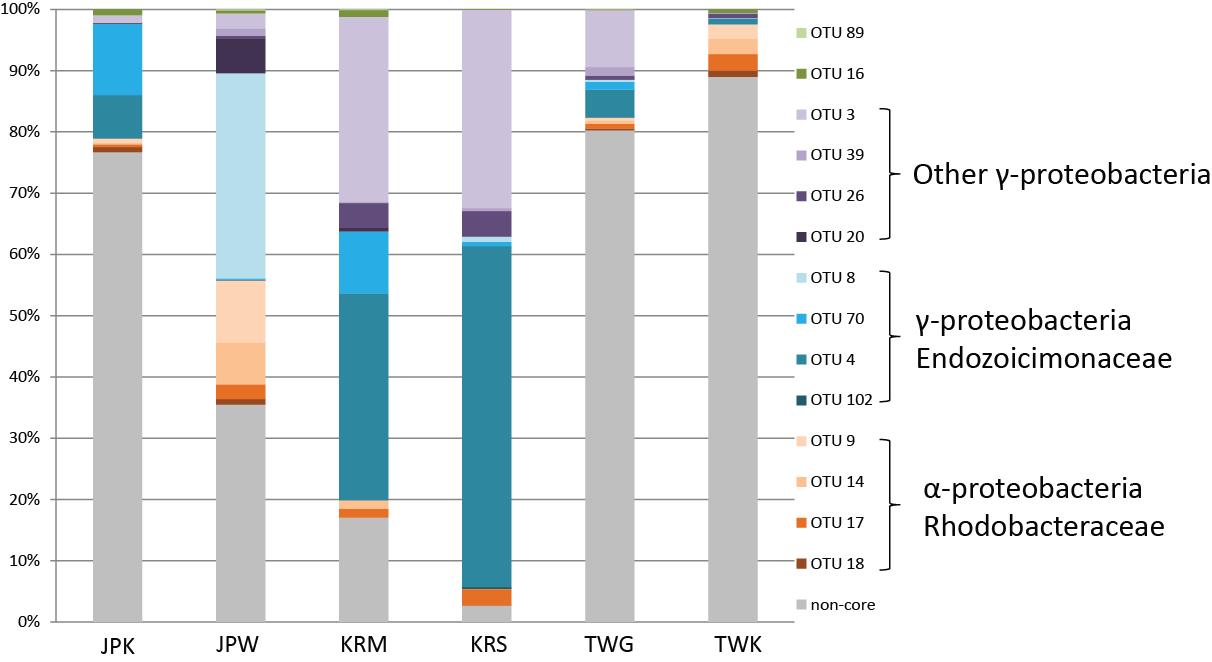


**Figure S3. The relative abundance of 14 core members in each location.**

Supplement: S3 Fig — Colors indicate OTUs. The S. gracillimum associated bacterial composition were denoted as TWG (Green Island, Taiwan), TWK (Kenting, Taiwan), KRM (Moonsum, Korea), KRS (Sungsan, Korea), JPK (Kochi, Japan) and JPW (Wakayama, Japan). (DOCX) [file pone.0183663.s003.docx]
